# Evaluation of Global RNA Amplification and Its Use for High-Throughput Transcript Analysis of Laser-Microdissected Endosperm

**DOI:** 10.1155/2007/61028

**Published:** 2007-04-10

**Authors:** Robert C. Day, Les McNoe, Richard C. Macknight

**Affiliations:** ^1^Department of Biochemistry, University of Otago, P.O. Box 56, Dunedin 9054, New Zealand; ^2^Genomics Facility, Department of Biochemistry, University of Otago, P.O. Box 56, Dunedin 9054, New Zealand

## Abstract

Laser microdissection (LM) provides a useful method for isolating specific cells or tissues from biological samples. Here, we adapted microdissection protocols to allow high-resolution transcript analysis of different tissues from developing *Arabidopsis* seed. Sufficient RNA (∼50 ng) was extracted from endosperm tissue for RT-PCR. However, to obtain enough RNA for microarray analyses, it was necessary to amplify the RNA. PCR- and IVT-based amplification methods were investigated and several important technical aspects of amplification were identified (such as target truncation and alterations in signal intensity). We found that when starting from only 50 ng of RNA, amplification methods based on PCR and IVT produced sufficient product for reliable microarray hybridizations, with two-round IVT giving the best results. Microarray analyses, using endosperm-derived RNA amplified by two-round IVT, reproducibly identified endosperm enriched marker genes. Thus, when combined with RNA-amplification protocols, LM is a robust and reliable technique for high-throughput tissue-specific gene expression analysis.

## 1. INTRODUCTION

Traditionally, to obtain enough material for global transcript analysis, whole organs or tissues have been used. However, by starting from a mixture of cells or tissues, key changes occurring in just one of the cell types may be obscured. To avoid this, approaches have been developed that allow high-resolution tissue sampling or enrichment for specific cell types [1]. In some cases, such as pollen grains or epidermal cells, the target cells are easily accessible for harvest or manual dissection. However, most cell types are embedded and require tissue digestion or microdissection to negate surrounding cell layers. Laser microdissection (LM) is an important method for obtaining individual tissues, cells, and even organelles for biochemical analysis. Originally developed for isolating cancerous cells from normal tissue [2], LM is beginning to be used in plant biology (reviewed by [3]). Compared to manual microdissection, modern laser-based systems are easy to use, highly reproducible, and can avoid direct contact with the biological sample [4]. LM samples have been used successfully to obtain DNA, RNA, proteins, and metabolites from a range of plant species and tissue types [5–7].

One disadvantage of isolating specific cell types is the relatively large amount of effort required to isolate a very small amount of material. In the cases of protein and metabolite analysis, microdissection is still a very inefficient approach for global analysis [6, 7]. However, RNA can be amplified; a process that can be carried out using a variety of methods [1]. The most common method of amplification is based on *in vitro* transcription (IVT) [8]. This linear amplification method involves producing double-stranded cDNA with a T7 priming sequence at the 3′ end. This template is then used by T7 RNA polymerase to generate copies of the cDNA template.

Compared to IVT-based methods, the exponential nature of PCR-based approaches enables much greater yields per round of amplification [9]. This means that far less starting material can be used to obtain enough target for high-throughput analysis, with just one round of amplification. For both IVT- and PCR-based methods, multiple rounds of amplification can be used to increase the yields when starting with very small amounts of RNA [10]. Hybrid methods have also been developed that use PCR and IVT amplifications, in an attempt to exploit the advantages of both [11, 12].

RNA amplification from very small amounts of RNA by IVT or PCR has several drawbacks. Reducing the starting amount of RNA to nanogram levels often leads to a reduction in the reproducibility of data. Extreme amplifications can also accentuate subtle technical biases giving loss of fidelity. However, since biases tend to be systematic, accurate expression data can be obtained if appropriate amplified controls are used [13]. For example, when comparing expression changes between samples, it is essential that all samples are similarly amplified.

*Arabidopsis* is being used as a model plant to study many aspects of seed biology; however, a major drawback is the very small size of its seeds. This causes numerous technical difficulties, especially when studying processes of early seed development. The endosperm appears to play several important roles during seed growth and development [14]. In many plant species, including *Arabidopsis*, the triploid primary endosperm nucleus undergoes several rounds of free-nuclear division, growing rapidly as a syncytium. Towards the end of this proliferative phase, three mitotic endosperm domains are established. The micropylar endosperm surrounds the embryo, the peripheral domain lines the wall of the developing embryo sac, and the chalazal domain develops adjacent to the vascular connection with the seed parent. The early proliferation of the endosperm is associated with final seed size and the alteration of the rate and duration of cell division in the endosperm has been proposed as a biotech strategy for altering seed size [15, 16]. Seed with severely defective endosperm cannot complete development which suggests a role for endosperm in supporting the formation and growth of the embryo [17].

LM provides an ideal tool for analyzing gene expression changes in specific cell types during the early stages of *Arabidopsis* seed development. The key to the success of such studies is the ability to isolate and amplify RNA from a small amount of laser-dissected tissue. Here, we present protocols for the isolation of RNA from different seed compartments of developing *Arabidopsis* seed and highlight important technical issues encountered when amplifying from small amounts of total RNA for array analysis. The protocols developed in this study were used to identify a list of approximately 2 700 genes that are expressed in proliferating endosperm 4 DAP.

## 2. MATERIAL AND METHODS

### 2.1. Plant growth

*Arabidopsis thaliana* plants were grown under lights in a growth room with 16-hour photoperiod and at 20°C. To produce seed of a known developmental stage, flowers were emasculated with fine forceps and manual pollinations were carried out two days after.

### 2.2. Harvest of siliques and RNA extraction for amplification

Multiple siliques corresponding to early and late stages of seed development were harvested into Eppendorf tubes and snap frozen in liquid nitrogen. Siliques from the early sample were added to the late sample at a ratio of 1 to 10 (based on fresh weight) to produce a 90% late sample. Early and 90% late tissues were ground to a fine powder in liquid nitrogen using a pestle and mortar. Total RNA was isolated from the samples using Concert Plant RNA Purification Reagent (Invitrogen, Carlsbad, Calif, USA) following the manufacturers' large-scale extraction protocol. Aliquots of these samples were further purified using Qiagen RNeasy columns and the manufacturers' optional on column DNase step was carried out.

### 2.3. Harvest of siliques for LCM tissue preparation

Siliques were harvested 4 days after pollination into precooled (4°C) farmers' fixative (3 : 1 mixture of ethanol : acetic acid). To enhance penetration of the fixative, the ends of the siliques were removed with a scalpel before the tissues were submerged. Samples were kept for 3-4 hours on an orbital platform at 4°C. The fixative was replaced with 95% ethanol for 30 minutes. The siliques were transferred into sample cassettes that were then submerged in absolute ethanol and were microwaved at 60°C for 10 minutes (Electron Microscopy Sciences EMS-820). Microwave treatment was repeated in two changes of isopropylalcohol at 70°C for 10 minutes and a third change at 74°C for 10 minutes. Cassettes were then transferred to molten wax and placed in a heated vacuum chamber. A vacuum was applied for 20 minutes then released. The vacuum infiltration of wax was repeated twice more using fresh wax. Siliques were released into molten wax and multiple siliques were aligned in single wax blocks and stored at 4°C with desiccant for up to two weeks before being sectioned using a rotary microtome (Leica Microsystems, Wetzelar, Germany) to produce 7 *μ*m sections. The sections were floated on distilled water and then 30% ethanol prior to attachment to superfrost plus slides (BDH/Merck, Damstadt, Germany). Slides were allowed to dry for 2 hours before storage with desiccant at 4°C.

### 2.4. LCM

Slides were dehydrated in xylene for 5 minutes and allowed to air dry in a flow hood for 5–10 minutes prior to LCM using the Arcturus LM200 Pixcell II system. This system enables specific regions of the tissue sample to be targeted microscopically through a plastic cap coated with a special thermoplastic film. The laser was then used to melt the film onto the targeted areas using a spot size of 7.5 *μ*m, power setting of 100 mW, and pulse durations of 1-2 milliseconds, depending on the ease of capture from each specific seed. Once cemented to the film, targeted tissues were removed from the rest of the section by lifting the cap vertically. Captures were collected onto Arcturus macrocaps. If nonspecific pickup was apparent a post-it note was used to remove the contaminating cells (3M, St Paul, MN, USA). The remaining tissue was sometimes scraped from the glass slide using a sterile flat-ended spatula, and was subsequently processed using equivalent protocols to the LCM material.

### 2.5. RNA extraction from tissues prepared for LCM

Total RNA was obtained from embryo, seed coat, syncytial endosperm, and the tissue scrape samples using the Picopure RNA isolation kit (Arcturus, Oxnard, CA, USA) with optional on-column DNase step (Qiagen, Valencia, CA, USA). The purified total RNA was quantified using the Ribogreen RNA quantification kit (Invitrogen) according to the manufacturers' instructions.

### 2.6. Conventional target labelling

Aliquots of 30 *μ*g total RNA were reverse transcribed and labelled using the Superscript Indirect labelling kit (Invitrogen).

### 2.7. Global transcript amplification by PCR

Aliquots of 50 ng total RNA were subjected to reverse transcription using superscript III reverse transcriptase (Invitrogen) and amplified dsDNA was prepared following the methods described by Petalidis et al. [18], with the exception that the entire cDNA sample was used in the amplification reaction. A test PCR is essential to avoid overcycling of the amplification reaction; however, this effectively doubles the amount of total RNA required to carry out RNA amplification. To determine the optimal number of cycles required for generating products during the exponential phase of the PCR reaction, PCR products from parallel runs were visualised after 15, 18, 21, and 24 cycles on a 1.2% agarose gel. When the yield of PCR product stops increasing with more cycles, the reaction has reached its plateau. The optimal number of cycles for the amplification is one cycle fewer than is needed to reach the plateau; in our case 20 cycles. Amino-allyl dNTPs were incorporated by Klenow and the free Cy dyes (Amersham Biosciences, Little Chalfont, UK) were coupled to the target dsDNA as described in the Superscript Indirect labelling kit manual (Invitrogen).

### 2.8. Global transcript amplification by IVT

IVT-based amplifications were carried out using the Message Amp II amino-allyl aRNA kit (Ambion Inc., Austin, TX, USA) following the manufacturers' instructions. Where appropriate, amino-allyl UTPs were incorporated into the IVT reaction and were coupled to Cy dyes (Amersham Biosciences) following the kits recommendations. All labelled aRNA targets were fragmented prior to hybridization using Ambion fragmentation reagent following the manufacturers' protocol (Ambion).

### 2.9. RT-PCR and qRT-PCR

The RNA or aRNA was primed with random hexamers and first-strand cDNA was synthesized using Superscript III (Invitrogen) according to the manufacturers' instructions. The amplification conditions for conventional PCR were as follows: 2 minutes at 94°C; 25–35 cycles of 30 seconds at 94°C, 30 seconds at 58, 30 seconds at 72°C; and 68°C for 2 minutes. The primers for *FWA*, *MEA*, and *AP2* are described by Kinoshita et al. [19, 20].

Real-time qRT-PCR was carried out using reagents from the LightCycler FastStart DNA Masterplus SYBR Green I kit (Roche, Penzberg, Germany) in 20 *μ*L volumes using a LightCycler 2.0 (Roche). The amplification conditions for qPCR were Denat: 95°C for 10 minutes; Cycling 94°C for 5 seconds, 58°C for 17 seconds, 72°C for 10 seconds (single acquire); Melt: 95°C for 0 second, 55°C for 20 seconds, 95°C for 0 second with ramp 0.2°C/s (continuous acquire); Cool: 40°C for 20 seconds. Quantification was carried out using Actin-2 as a reference gene (At3g18780). Actin-2 was selected since it was a control spot on our microarrays. The differential expression observed across the 17 spots per slide representing Actin-2 in our normalized data was 1.004 indicating equivalent expression in both types of biological sample used to query the arrays and used for qRT-PCR. Reaction products were confirmed by melting curve analysis and by running out the product on a 1.2% agarose gel. Primer sets for qRT-PCR validation experiments were designed to span at least one intron so we could easily observe PCR products generated from genomic DNA contamination. For each primer set, a no-template water control was also used.

The primers used for qRT-PCR of *FWA* and *MEA* are described by Kinoshita et al. [19, 20] and the *FIS2* primers were FIS2-RT-R 5′-agatctcctggcgactaac-3′, and FIS2-RT-F 5′-tattagcgaacgcctgagac-3′. Actin primers were Q-RT Actin_F 5′-cgctctttctttccaagctcat-3′ and Q-RT Actin_R 5′-tcctgcaaatccagccttc-3′. TT8 primers were TT8-QRT-F 5′-ctgatcttcatattgaatcaaccca-3′ and TT8-QRT-R 5′-gtgtgacatgagaagtgttgttac-3′. FUS3 primers were FUS3-QRT-F 5′-tcatggtctgcagctaggtga-3′ and FUS3-QRT-R 5′-tacttcttcttcttccgatgcttt-3′. At5g16780 primers were CAS5G-QRT-F 5′-gtgggtttgcgactgttg-3′ and CAS5G-QRT-R 5′-gagtttccgggctctgatt-3′. At1g48630 primers were CAS1G-QRT-F 5′-aagtctgttgttgaggatttgaag-3 and CAS1G-QRT-R 5′-ttccatctgcactccagtt-3′.

### 2.10. Array hybridization

*Prewash and BSA prehybridization*: slides were prewashed in 0.2% SDS for 2 minutes and then washed twice in MilliQ (MQ) water. The slides were then placed in a 50 mL Falcon tube containing fresh MQ water and were incubated in a water bath for 20 minutes at 50°C. Slides were then prehybridized in buffer containing 4x SSC, 0.5% SDS and 5% w/v bovine serum albumin (BSA, Sigma) at 42°C for 45 minutes. Slides were then washed 5 times with MQ water at room temperature to remove BSA residue. Prior to hybridization, the slides were dried by centrifuged at 2,500 xg.

*Hybridization*: hybridizations were carried out under LifterSlips (Erie scientific, Portsmouth, NH, USA) placed over the region of the slide containing the array. A mix of SlideHyb buffer 1 (Ambion) and the labelled probes were then injected under the LifterSlip. The arrays were then placed in a humidified hybridization chamber (Corning, New York, NY, USA) and were hybridized over night in a 55°C water bath.

*Posthybridization*: the slides were removed from the hybridization chamber and were washed at 55°C for 8 minutes with agitation in 50 mL Falcon tubes containing 2x SSC, 0.1% SDS. The slides were then moved to new 50 mL Falcon tubes and were washed with constant agitation in 1x SSC, 0.1% SDS at room temperature for further 6 minutes. This procedure was repeated with a solution containing 0.5x SSC and then 0.1x SSC before the slides were dried by centrifugation at 5800 rpm.

*Scanning*: slide scanning was carried out using an Axon 4000 B dual-color scanner running GenePix 4.1 software (Axon instruments/Molecular Devices Corporation, Sunnydale,CA, USA).

### 2.11. Data normalization and analysis

Genepix files were normalized by implementing elements of the SNOMAD gene analysis tools (http://pevsnerlab.kennedykrieger.org/snomadinput.html) at a local level using an SPLUS script. This involved global mean normalization, local mean normalization across the array surface, and local mean normalization across the element signal intensity. Both high and low power scans were obtained for each hybridization. Data from high and low scans were normalized independently. When calculating correlations, the number of differentially expressed transcripts and the direction of change, the saturated spot data was replaced with data from the low scan. Any spots that had intensity values less than 3x the mean background in both channels or were only represented in a single replicate were discarded from this analysis. Genes that had a signal above the trim level in only one channel remained in the analysis but had a value of 100 intensity units interpolated for the low signal to reduce the effect of very low intensity features on expression ratios. Interpolation introduced large amounts of variation for transcripts that gave very low signal. Data was normalized without interpolation for use with genes that had very low signal, for example, *FIS2*. When interpreting the data with regard to movement of intensity values within the population or loss of signal based on probe design, we only considered the high intensity scan and the data was not trimmed based on intensity.

The microarray data has been deposited at NCBI GEO (http://www.ncbi.nlm.nih.gov/geo) accession number GSE6703.

## 3. RESULTS AND DISCUSSION

### 3.1. Development of tissue preparation protocols for laser capture microdissection of Arabidopsis seed

**Figure 1:**
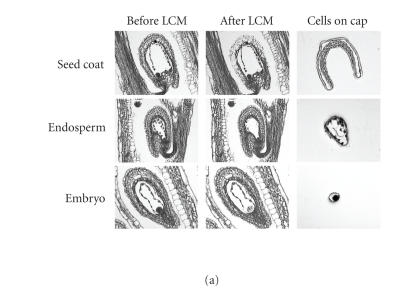

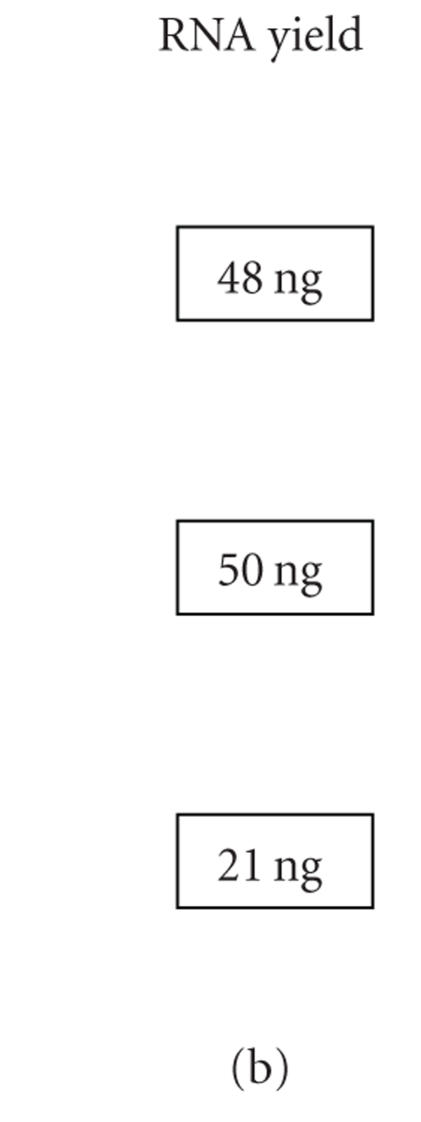

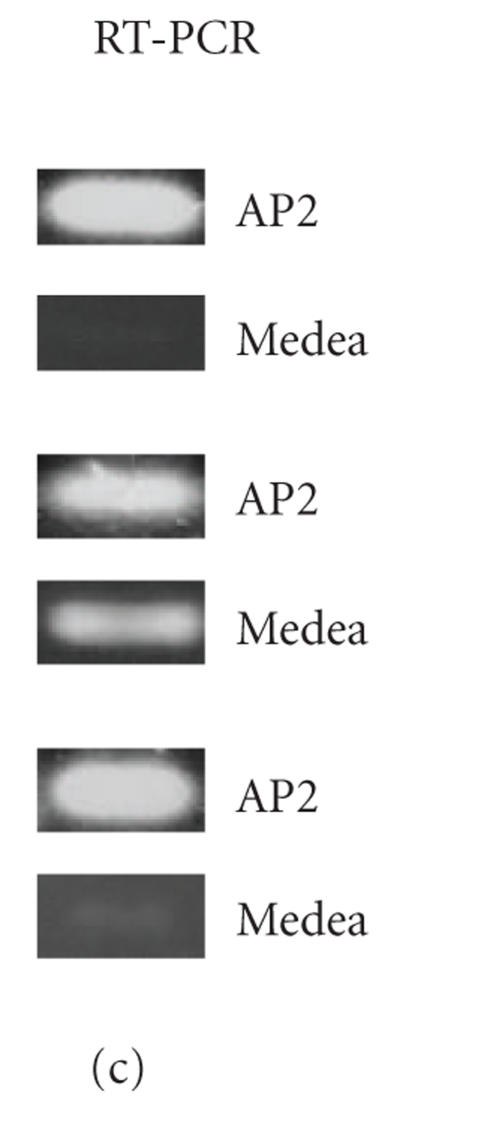
Laser capture microdissection (LCM) of seed for transcript analysis. (a) Examples of laser capture showing that uncontaminated seed coat, endosperm, and embryo tissues can be isolated. (b) The boxes show the mean yield of RNA extracted from the different seed compartments, SE were ±9.6, ±6.7, and ±3.1 for seed coat, endosperm, and embryo, respectively (*n* = 6). (c) RT-PCR of *AP2* and *MEA* from LCM-derived (top to bottom) seed coat, endosperm, and embryo.

Laser capture microdissection (LCM) is a form of LM that relies on the adhesion of only the targeted cells to a thermoplastic film attached to a plastic cap [2]. We developed tissue preparation protocols for LCM of immature *Arabidopsis* seed using Farmer's fixative based on recommendations of Kerk et al. [5]. Other groups have successfully used acetone or microwave fixation in the absence of chemical fixative to carry out transcript analysis on plant material [21, 22]. However, our preliminary studies indicated that Farmer's fixative gives the most consistent penetration of the fixative into *Arabidopsis* silique tissues and enables good histology and yields of total RNA. Using Farmer's fixative, our tissue fixation period (3-4 hours) was shorter than protocols using acetone (overnight). We also used the heat generated by a microwave to aid the diffusion of subsequent solvent washes and the infusion of wax under vacuum, further reducing the processing time. This enabled silique material to be processed from harvest to wax block within 9 hours. Figure 1(a) shows examples of LCM isolations of seed coat, endosperm, and embryo tissue from developing siliques. LCM removed the majority of the target tissue and only the desired cells were visible on the LCM cap (Figure 1(a)). Fixed samples were aligned in the wax so that approximately six siliques were within the footprint of an LCM cap. This maximized the number of seeds that could be targeted onto each cap. Three serial sections were mounted onto a single glass slide, enabling the caps to be rapidly repositioned over each serial section in turn if target tissue was limiting (as was the case for embryo samples).

### 3.2. RNA extracted from microdissected seed is suitable for transcript analysis

As well as allowing sufficient histological resolution, the tissue-processing regime must be conducive to subsequent extraction of RNA of sufficient quality and in amounts suitable for meaningful analysis. We used the Arcturus Picopure RNA isolation kit with an on-column DNase treatment to isolate total RNA. In an alteration to the kit protocol, we routinely peeled the thermoplastic film from LCM caps using fine forceps and submerged these in extraction buffer. This allowed multiple caps to be extracted in the same tube. By combining two peels containing isolated material from multiple seeds, we were able to obtain 20–60 ng of total RNA per extraction, depending on the seed compartment targeted, for example, approximately 50 endosperm fragments were obtained per peel enabling 100 fragments to be used for RNA extraction (Figure 1(b)). The RNA yields were quantified using the Ribogreen RNA quantification kit (Invitrogen).

As with most studies of this kind, we assessed the quality of the extracted RNA by generating cDNA templates from unamplified samples and conducting RT-PCR [5, 21, 23, 24]. We also used small aliquots of aRNA produced from one round of IVT amplification for similar analysis (10–15% of total product). The cDNA yield from the aRNA increased our effective yield of experimental material and would be sufficient to carry out several hundred tissue-specific PCR reactions.

**Figure 2:**
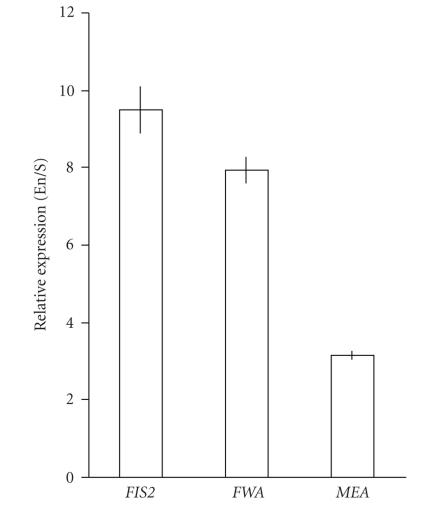
RNA derived from LCM isolated endosperm is enriched for endosperm-specific transcripts. Real-time quantitative RT-PCR indicating enrichment of *FIS2*, *FWA*, and *MEA* transcripts in LCM endosperm samples relative to that in the silique tissues remaining after microdissection. Expression was calculated relative to actin. EN/S indicates that the value obtained for the endosperm (En) was divided by the value obtained for the silique tissue scrape (S) to obtain the value for relative expression. Error bars represent the SE when *n* = 9.

Our lab is interested in identifying genes that are important during early endosperm development. To identify transcripts expressed specifically in the endosperm, we needed to ensure that the other seed compartment samples had minimal endosperm contamination and our endosperm sample was suitably enriched. To investigate this, we carried out RT-PCR on LCM-derived samples. As shown in Figure 1(c), AP2 was amplified from all samples using conventional RT-PCR, consistent with its reported expression throughout the early seed [19], and as expected *MEA* expression was relatively strong in the endosperm and weak in the embryo (globular to mid-heart) [25]. We used aRNA from a single round of IVT amplification to assess the enrichment of endosperm expressed transcripts by real-time qPCR. We calculated the levels of expression (relative to actin) of two endosperm-specific genes, *FWA* and *FIS2*, and *MEA* from both LCM-derived endosperm cDNA (4 DAP) and a similarly amplified tissue scrape cDNA. The scrape sample was derived from the tissues remaining on the slide after microdissection of the endosperm. Not all seed tissues enabled unambiguous identification and unimpeded access to endosperm tissue, thus the tissue scrape retained a proportion of endosperm material. Results indicated a strong enrichment for endosperm (Figure 2), such that the endosperm-specific genes *FIS2* and *FWA* reported 9.5-fold and 8-fold enrichment, respectively. *MEA* reported a 3.5-fold enrichment that reflects its simultaneous expression in the embryo [25, 26].

Taken together, our PCR analysis indicated that the RNA obtained using LCM generated expression data that was consistent with the expression of known marker genes and that the cell types were well separated.

### 3.3. Experimental design for the comparison of amplification methods using oligonucleotide microarray

Our aim was to identify which standalone amplification protocol performed best in our lab when the starting amount of total RNA was limited to 50 ng. While other studies have compared amplification protocols, few start with such low amounts of total RNA [10, 13]. In addition, most of these studies use ultra-high-quality RNA bought commercially or isolated from cell lines [27–33]. Since the quality of extracted RNA is very often tissue-dependent and will affect the amplification efficiency, we decided to use total RNA extracted from whole siliques; better representing tissues targeted by LCM. We set out to compare two basic methods of RNA amplification, based on IVT and PCR. Preliminary amplifications from LCM prepared samples indicated that two rounds of IVT or a single round of PCR were required to generate enough aRNA or cDNA, respectively, for hybridization to long oligonucleotide arrays (data not shown).

RNA was isolated from siliques during early (2–5 DAP) and late (6–10 DAP) stages of development. To help identifying major alterations in the array data due to our target preparation protocols, the late stage RNA sample was spiked with 10% early stage sample (90% late). In the absence of compression, the signals from the genes only expressed in the early sample should not be more than 10-fold higher than those obtained from the late sample. Conversely, the late sample should display a full range of differential expression. This design should result in the unamplified MA plots (plots of differential expression against intensity) having a distinctive asymmetrical shape.

Our study compared unamplified samples and three RNA amplification regimes; one-round IVT, two-round IVT, and a PCR-based method. The unamplified treatments used 30 *μ*g of total RNA followed by conventional labelling and hybridization. The one-round IVT amplifications were made from 5 *μ*g of total RNA (a standard labelling procedure in many microarray experiments). The two-round IVT and PCR regimes started from 50 ng of total RNA and would therefore be appropriate for use with LCM samples. The unamplified treatment involved duplicate hybridizations of the 90%-late sample against our early sample. For each of the amplification regimes, a single amplification of the 90%-late sample was hybridized against two independent amplifications of our early sample. In all hybridizations the early and 90%-late samples were consistently labelled with Cy5 and Cy3, respectively. This consistent labelling strategy for all 8 slides reduced variation between the microarrays due to dye bias and enabled us to focus on the variation arising from duplicate amplifications of a single early silique sample.

### 3.4. Reproducible microarray results can be obtained using amplified RNA

The RNA yields after amplification are given in Table 1. We aimed to generate 10–20 *μ*g of aRNA using IVT and 1–5 *μ*g of cDNA using PCR; such that we could comfortably perform duplicate hybridizations from each sample. When starting with 50 ng of RNA, the desired amount of aRNA/cDNA was obtained after either two rounds of IVT (103 *μ*g aRNA) or a single round of PCR amplification (10 *μ*g), respectively (Table 1). IVT-based amplification on our LM samples generated aRNA with a similar efficiency to that obtained from a conventional tissue sample (Table 1).

The reproducibility and fidelity of the amplification methods were assessed by microarray analysis using long oligonucleotide arrays based on the Operon V1.0 *Arabidopsis* set. The emergence of extremely high signal for some array elements post amplification (hotspots) has been described in the literature [34] and was countered in this study by carrying out both high and low power scans for each hybridization. Data from high and low scans were normalized independently and the saturated spot data replaced with data from the low scan. This enabled the highest possible number of data points to be included in our analysis and negated any issues due to alteration of signal spread due to different amplification regimes. In instances where data from the combined scan set might introduce artefacts into our analysis, we considered only data from the high power scans.

**Figure 3:**
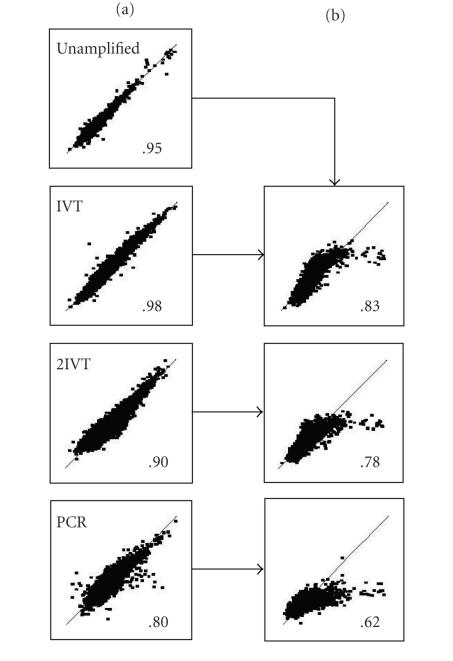
Reproducibility and fidelity of RNA amplification. (a) Scatter plots of the Log_2_ expression ratios from two hybridizations using duplicate targets prepared independently from the same samples plotted against each other. Target preparation regimes are given within the top-left corner of the individual plots. (b) Comparison of the Log_2_ expression ratios obtained from the amplified samples (*y*-axis) to the Log_2_ expression ratios from the unamplified samples (*x*-axis). Numbers contained within each plot represent the Pearson correlation * (r)*. Line represents *r* =1.

To assess the reproducibility of our methods, we plotted the duplicate data obtained for each target preparation regime (Figure 3(a)). The between-replicate reproducibility of the unamplified samples was high with a Pearson correlation of *r* = .95. The single-round IVT amplification gave an even higher between-replicate correlation (*r* = .98), a phenomenon that has been reported by others [35, 36]. The extreme amplifications also showed high between-replicate correlations of *r* = .8 and *r* = .9 for PCR and 2IVT, respectively. Puskas et al. [37] also found a higher correlation with IVT than with PCR, when the two amplifications were compared directly. Although our PCR and 2IVT correlations were somewhat lower than the more conventional target preparation strategies, they are consistent with other studies using RNA samples diluted down to tens of nanograms (*r* = .87–.94; [38, 39]).

In summary, amplification of 50 ng of RNA by either two rounds of IVT or PCR produced sufficient product for reproducible microarray hybridizations.

### 3.5. Amplification affects the profile of the RNA population

**Figure 4:**
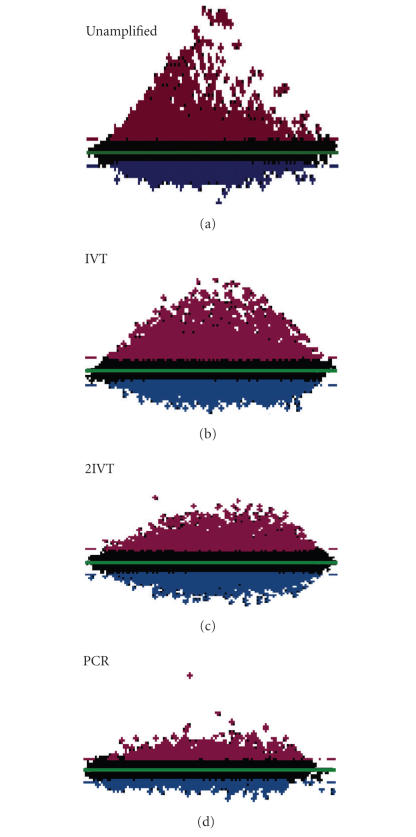
Relative expression and intensity are altered with amplification. Representative MA plots for the different target preparation regimes using normalized data from the high-intensity scans (Log_2_ expression ratio on the *y*-axis is plotted against the fluorescent intensity on the *x*-axis). Transcripts preferentially expressed during late seed development are shown in red; transcripts preferentially expressed during early stages of seed development are shown in blue, and unchanged are shown in black.

To determine if the different amplification methods fundamentally altered the global representation of the transcript populations, we first considered variations in the MA plots. These plots graph intensity on the *x*-axis versus Log_2_ of the differential expression on the *y*-axis. Our experimental design generated the expected asymmetrical form when the unamplified data were plotted in this way (Figure 4). All amplification regimes deviated from this pattern and appeared to compress the differential signals reported in the unamplified dataset. The magnitude of compression was (from lowest to highest) IVT, 2IVT, and PCR. The compression comprised a more reduced differential in the direction of the 90%-late sample than that observed in the early sample. This suggests that highly differential values were more affected than moderate fold changes. This was also evident in the correlation plots drawn between the amplified data and the unamplified data, where the most differential signals fell away from the *r* = 1 line (Figure 3(b)). The amplification regimes also reported a more consistent spread of intensity values (Figure 4).

**Figure 5:**
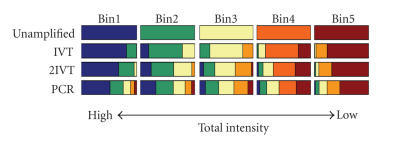
Effect of amplification on relative intensity values within the target population. Total intensity (Cy3 intensity + Cy5 intensity) values for each gene represented in all datasets were obtained from high-intensity scans. Data points were then sorted based on intensity in the unamplified dataset and were divided into five bins containing equal numbers of genes. Genes within each bin were differentially color-coded. The genes were then resorted in the same way for each treatment but retained the color code from the unamplified sample. Graph shows the changes in relative total intensity that occurred following amplification.

To assess the affects of amplification on relative transcript abundance, we calculated the total intensity (sum of red and green signals) for each gene within each treatment. These values were placed in rank order and were split into five groups based on intensity. The transcripts contained within each intensity bin were then tracked back to see which intensity groups the transcript originated from in the unamplified samples. Amplification caused alterations in the relative intensity of a subset of genes; an effect that was most pronounced with extreme IVT- or PCR-based amplifications (Figure 5). PCR was the worst affected, with many transcripts moving into widely different intensity groups.

Our amplified data gave good positive correlations to the unamplified samples (*r* = .62–.83) and independent studies describe similar values [40, 41]. Many studies that assess fidelity of amplification have been carried out using Affymetrix Genechip technology [10, 13]. These studies tend to give higher correlations between amplified samples, than those obtained here using academic printed slides, a situation that is perhaps partly attributable to issues of manufacture. However, Genechip technology can exclude up to 50% of the genes detected by long oligonucleotide-based arrays, based on their low signal intensity. This effectively excludes transcripts with low correlations from the analysis [42]. Furthermore, in the majority of Genechip studies, the conventional target preparation method used is a mild form of IVT based-amplification. If we compare our two-round IVT amplification to our IVT labelled sample our correlation increases from a Pearson correlation of *r* = .78 to .9. Conversely, our PCR amplified dataset had a lower correlation to the IVT labelled sample than it did to the unamplified dataset. This indicates that the different amplification strategies can introduce different distinct biases. Other aspects of target preparation can also introduce sources of variation that can affect the correlation between datasets. Reverse transcriptases can vary in their ability to yield useful amounts of cDNA from limiting amounts of RNA [43] and in their ability to generate cDNA that largely maintains the length of the original transcript, an important issue when using oligonucleotide-based microarrays (see later). Comparison of DNA hybridizations (unamplified and PCR), with fragmented aRNA hybridizations (IVT), may also introduce variation due to different hybridization kinetics [44].

**Figure 6:**
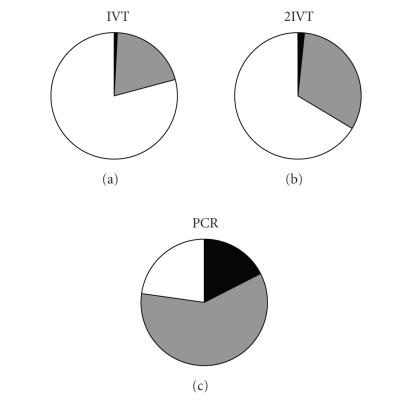
Effect of amplification on the direction of differential expression. Pie charts indicating the proportion of genes, in each of the three amplification regimes, show the same direction of differential expression as the unamplified samples. The set of genes that were 2-fold or more differentially expressed in the unamplified sample were tracked in amplified data. The genes were divided into 3 groups: white, the directionality was maintained and the magnitude of the change remained above the 2-fold threshold; gray, the directionality of the change was conserved but the magnitude dropped below the 2-fold threshold; and black, the directionality of the change was not conserved.

Despite obvious alterations in the observed signal from our hybridizations after amplification, we wanted to assess whether the direction of differential expression was consistent. The genes that reported a two-fold differential expression in the unamplified treatments were tracked in the amplified data. It became apparent that while many genes reported an altered level of differential expression, the majority of these genes gave a consistent direction of change, irrespective of the method used or extreme nature of the amplifications (Figure 6). Considerable variation in fold change has been observed in microarray data sets, compared to real-time PCR [45–47]. Often microarray data changes are compressed and this phenomenon is more apparent in amplified samples [48]. Ignoring or relaxing the fold change cutoff and using statistical methods can to a large extent eliminate problems due to compression. Jenson et al. [48] found that by dropping the threshold from 2 to 1.5, they got an increased number of differentially expressed genes common to both cDNA (unamplified) and aRNA (amplified) generated microarray data sets.

Overall, our results suggest that while the different amplification methods report variable differential expression for the same gene, they are reasonably reliable at indicating if a gene is up- or down-regulated. Thus, in instances where compression is likely, such as with extreme amplifications, it might be advisable to base interpretation of data mainly on the direction of change.

### 3.6. Different amplification methods identify different differentially expressed genes

**Figure 7:**
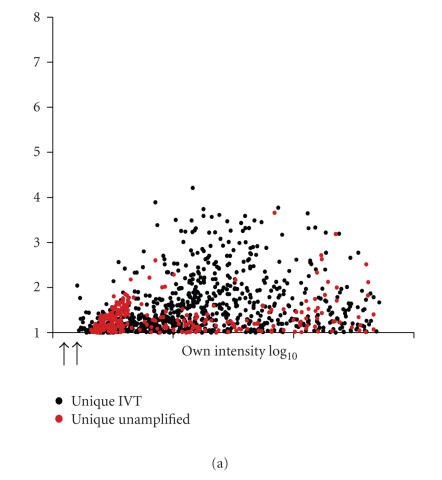

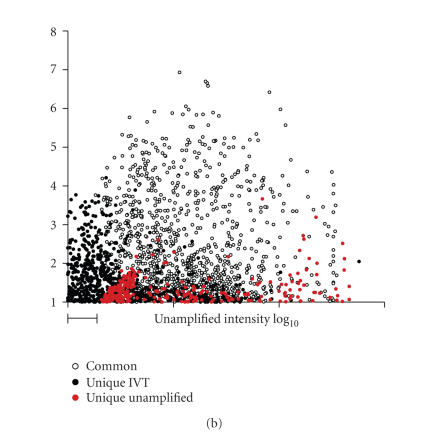
Analysis of low-copy transcripts is enhanced using IVT. The relative expression (Log_2_ ratio) on the *y*-axis plotted against the total intensity values (sum of both channels) on the *x*-axis. (a) The relative expression of the unique IVT and unamplified differentials plotted against their own total intensities. The data were similarly distributed to the right of the x3 background intensity cutoffs indicated by arrows under the *x*-axis. (b) The relative expression of the unique IVT and unamplified differentials plotted against the total intensities from the unamplified hybridization. A large number of the unique IVT differentials represented transcripts that described low-intensity positions in the unamplified dataset (see bar). Red and black circles represent differentially expressed genes unique to the unamplified sample or the IVT sample, respectively. White circles represent differentially expressed genes identified in both the unamplified and the IVT datasets.

Our experiments were not designed to give precise data about the differential expression of specific genes. Nevertheless, large fluctuations in the numbers of transcripts reporting two-fold differential expression were apparent, and worthy of comment. The different amplification regimes reported a sizable percentage (6%–12%) of unique differentials (Table 2(a)). Furthermore, the mild IVT amplification reported more genes with a differential expression (>2-fold) than all other treatments, including the unamplified data (Table 2(b)). When we plotted the fold change of the unique IVT and unamplified differentials against their total intensities, the data were similarly distributed to the right of the 3x background intensity cutoff (Figure 7(a)). However, when we plotted the unique IVT differentials against the intensity values from the unamplified data, it became apparent that the majority of the unique IVT differentials represented transcripts of low intensity in the unamplified dataset (Figure 7(b)). This indicated that in some instances, amplification can increase the absolute signal intensity allowing more differentials to be identified. The emergence of such data from low intensity background probably represents a systematic bias in the amplification of specific transcripts. Nevertheless, several studies have verified that differentials specifically identified in amplified samples can be validated by other methods, such as real-time PCR [31, 49, 50].

Taken together, these data suggest that even if RNA yields were conducive to conventional cDNA hybridizations, the optimum strategy for compiling a list of differentially expressed genes would be several hybridizations using a combination of different target preparation regimes.

### 3.7. Extreme amplification can truncate representative aRNA and cause subsequent reduction in signal on long oligonucleotide arrays

**Figure 8:**
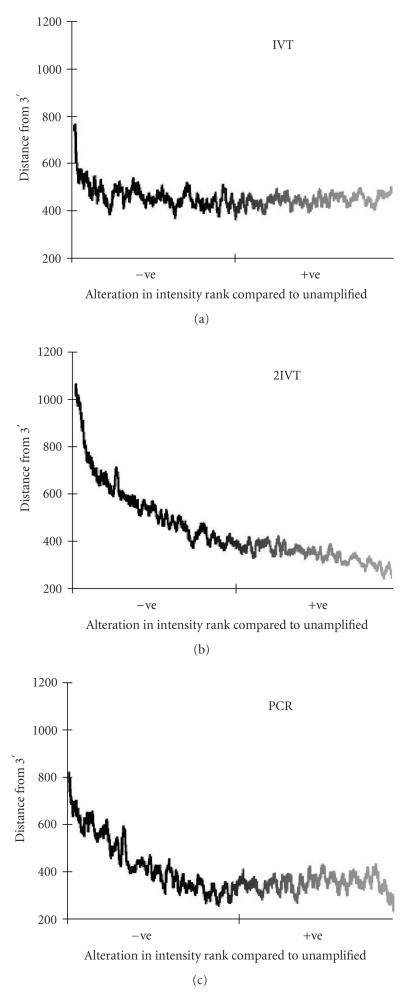
Reduced signal intensity correlates with a more 5′ position being targeted by the microarray oligonucleotides. The position of the microarray probe oligos sequence within the annotated mRNA from Genbank was plotted against the moving average of the spot intensity, relative to the unamplified sample. +ve and −ve represent an increase or decrease in relative intensity, respectively. The moving average was calculated from 50 data points.

The second round of IVT uses a random primer for first-strand cDNA synthesis, a process that may lead to truncation of the representative target molecules [51]. To assess the effect of the second-round IVT on our data, each treatment dataset was ranked based on total intensity and the ranks for each probe were compared to rankings in the unamplified data.The relative ranking was calculated such that positive or negative changes in rank indicated an increase or decrease in relative signal within the target population, respectively. This change in apparent signal was then plotted versus the distance of the corresponding target probe sequence from the 3′ end of the annotated target transcript (Figure 8). Our microarrays were printed using the Operon version 1.0 *Arabidopsis* oligonucleotide set. Whilst these oligonucleotides were designed to the 3′ region of genes, their position varies with regard to distance from the polyA tail. The plot shown in Figure 8 indicated that more genes were being lost from our dataset if extreme amplifications were used for target preparation, and that this correlated with the design of some of the microarray oligos being more 5′ biased than others. Although not as severe as the target truncation introduced during the random priming of the second round of IVT, PCR amplification also has detrimental affects on target length (Figure 8). The template switching method utilized by the PCR regime integrates primer sites into both the 5′ and the 3′ ends of the representative cDNA templates that should negate issues of 3′ bias. However, other studies have also found that PCR can generate products shorter than those obtained from one round of IVT [52]. This is presumably due to differences in the abilities of different reverse transcriptases to generate full-length cDNA.

The latest version of the Operon *Arabidopsis* oligonucleotide set should be less sensitive to target truncation, as it is more 3′ optimized. In addition, not all array types will be as affected by target truncation. Arrays based on printed full-length cDNAs, tiling arrays, and conventional Affymetrix Genechips will have better 3′ representation than our arrays. The long oligonucleotide arrays used in this study generally had a single probe representing each transcript. This is in contrast to Affymetrix Genechips, which have several probes per transcript that are 3′ optimized. In instances where more than one round of IVT are used, this can be advantageous. Casson et al. [53] generated target populations from LCM harvested embryo cells prepared for Genechip analysis by three rounds of IVT-based amplification. They also found a reduction in 3′ representation for some transcripts, but by considering fewer representative probes in their analysis they were able to identify a larger number of embryo-expressed genes [53].

### 3.8. Tissue-specific microarray analysis of endosperm

We have shown here that the two-round IVT-based amplification provided good between replicate correlations and that it was the extreme amplification method that best represented our unamplified data. However, since this data was obtained using 50 ng of conventionally purified RNA rather than RNA obtained from a microdissected sample, we investigated whether microarray data produced u sing LCM-derived RNA also gives highly reproducible results. We used a two-round IVT-based amplification to obtain expression profiles from three replicate endosperm samples microdissected from sections of 4 DAP siliques and from the tissues remaining post-microdissection scraped from one of the sample slides. The tissue scrape aRNA was used as a universal control for all endosperm hybridizations. This approach was used to minimize alterations in the measured differential expression due to differences in RNA quality [54].

### 3.9. Reproducibility of array data obtained using LCM endosperm

**Figure 9:**
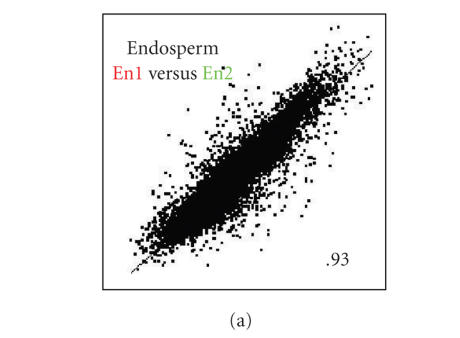

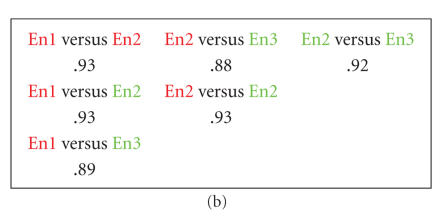

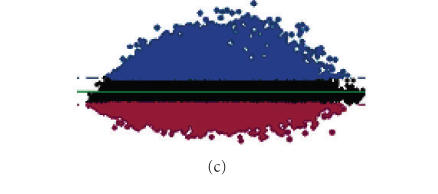
Microarray analysis of microdissected endosperms is reproducible. (a) A representative scatter plot of expression data (Log_2_ ratio) from two replicate “endosperm versus silique tissue scrape” hybridizations. This combination compares data involving two biological endosperm replicates that were also dye swaps. (b) Pearson correlations between the four endosperm hybridisations. (c) A representative asymmetrical MA plot obtained from an endosperm versus tissue scrape hybridization. En1–3 represent hybridizations involving the three different endosperm samples and the red or green font color denotes labelling of the endosperm samples using Cy5 or Cy3, respectively.

The silique sample (tissue scrape) included a proportion of endosperm such that the MA plots for hybridizations between silique tissue scrapes and LM endosperms described an asymmetrical form similar to our previous amplification experiments (Figure 9(c)). Pearson correlations between our replicates were high (0.88–0.93) indicating that the microarray data produced using our LM-derived RNA generated highly reproducible results. The lowest correlations were obtained from comparisons between biological replicates that incorporated dye-swaps (Figures 9(a) and 9(b)). 18 220 unique probes gave signal higher than two-fold background and were represented in at least three of the four hybridizations. *P* values were calculated to assess the variation of the signal across the four endosperm hybridizations. Twelve thousand seven hundred and ten (12 710) probes gave consistent signal with *P* values of <.05. Of this list, approximately 5 000 probes allocated to individual loci were differentially expressed in the endosperm sample using a 1.5-fold cutoff or approximately 2700 probes if a 2-fold cutoff was used (listed in the Supplementary Table 1 available at doi:10.1155/2007/61028).

### 3.10. The LM endosperm array data does not appear to be contaminated with seed coat- or embryo-specific genes

During LCM of endosperm from seed at 4 DAP, many individual microdissections were performed. Tissues such as the inner endothelium of the developing seed coat and the developing embryo are immediately adjacent to the endosperm and are the most likely contaminants of our endosperm samples. Few genes have been characterized as being preferentially expressed in the inner endothelium of the seed coat at 4 DAP. However, two such genes TT8 and BAN [55, 56] gave consistent signal in our array data and were used to assess seed coat contamination (TT8 was also represented on our slides by two independent probes). Both these seed coat markers were excluded from our endosperm expressed list even using a 1.5-fold cutoff, indicating that there is little seed coat contamination of our endosperm LM samples.

To screen our data for embryo contamination, we compiled a list of sixteen embryo-expressed genes from the literature. In an LCM study that targeted *Arabidopsis* embryos at early stages of development, Casson et al. [53] validated their embryo Genechip experiments using a list of embryo-expressed genes. This study also generated several reporter-GUS lines that provided evidence for the expression of several additional genes in early embryos. Thirteen of the sixteen embryonic markers were excluded from our endosperm-expressed list using a 2-fold cutoff. Of the three embryonic markers present in the endosperm expressed list, *LEC1* has been reported to be expressed in both the embryo and the endosperm at heart stage by *in situ* hybridization [53, 57] and there is evidence from an At1g48630 promoter-GUS reporter lines that this gene is also expressed in both embryo and endosperm [53, 57].

The expression of the third embryonic marker, *FUS3*, in the endosperm at 4 DAP is inconsistent with the genes characterization in the current literature. Both *FUS3* promoter-GUS [58] and FUS3-GFP translational fusions [59] give strong and highly specific activity in globular embryos. Expression of *FUS3* in the embryo at heart stage (approximately 4 DAP) then becomes more restricted to the outer protodermal tissues. Interestingly during the later stages of development, *FUS3* expression is reported to be strong in the endosperm tissues that form the aleurone layer. It is possible, therefore, that at 4 DAP, *FUS3* is starting to make the transition from embryonic-specific expression to a more widespread expression pattern. Furthermore, the strong signal from the embryo may obscure a lower more diffuse activity in the endosperm. Images presented for *FUS3* promoter-GUS fusions by Tsuchiya et al. [58] appear to be consistent with this hypothesis since the endosperm visible in the heart stage sample (limited to the embryo surrounding region) appears to show GUS activity. Thus, our endosperm-expressed list does not appear to be contaminated with any genes that are unequivocally expressed specifically in the embryo.

To further validate our data, the expression of the two embryonic marker genes not described in the literature as endosperm expressed (*FUS3* and At4g48630) were validated by real-time PCR. Real-time PCR of the embryonic marker At5g16780 and seed coat marker TT8 were also used to clarify the expression of marker genes close to the 1.5-fold cutoff (Table 3). For the embryonic markers (FUS3, At1g48630, and At5g16780) real-time PCR gave largely equivalent values to those obtained from the array experiments. For TT8, the differential expression observed by real-time PCR was slightly higher in the endosperm direction than that observed in our array data and took the transcript over the 1.5-fold threshold to a value of 1.66. Since both TT8 and At5g16780 marker genes gave some evidence of being over 1.5-fold differentially expressed in the endosperm, it was thought prudent to limit the endosperm-expressed list to genes above a 2-fold cutoff (Table 3).

### 3.11. Known endosperm-expressed genes are present in the LM endosperm data

To confirm that our approach could detect differential expression of known endosperm-expressed genes, we identified a number of genes from the literature that have been described as endosperm-preferred or endosperm-specific. Tiwari et al. [16] recently identified a number of candidate genes that gave strong evidence for endosperm-preferred expression. A selection of these was used for validation by promoter-GUS fusions [16]. Four of the genes identified as having upstream regions that can drive GUS expression during the early stages of endosperm development gave consistent signal in our LM data (At5g46950, At5g07210, At5g39260, At2g41000). However, only At5g07210 (ARR21), a two-component response regulator, was identified as having endosperm-preferred expression from our 4 DAP data.

Interestingly, expression of both At5g46950 and At5g39260 in the AtGenExpress Genechip developmental series [60] is only evident in siliques at 3 DAP, when the seed contains globular stage embryos, and not in siliques at 4 DAP (heart stage). This suggests that the LM endosperm data is extremely accurate with regard to developmental stage. Further evidence for this is provided in a study that examined the expression of isopentenyltransferases (IPT) genes by promoter-GUS reporter constructs. *IPT* genes catalyze the rate limiting step of cytokinin biosynthesis in *Arabidospsis*, and Miyawaki et al. [61] found that *IPT4* and *IPT8* were expressed predominantly in the chalazal endosperm of developing seed. *IPT8* had a slightly more extensive expression pattern in the endosperm and persisted until late heart stage, whereas *IPT4* was very specific to the chalazal cyst and the activity in IPT4-GUS transformants disappeared prior to heart stage. The inclusion of *IPT8* in our endosperm-preferred list (and the exclusion of *IPT4*) is consistent with precise sampling of the endosperm at 4 DAP when the seeds are at the early to mid heart stages of development.

At2g41000 encodes a DNAJ heatshock protein that describes a range of different expression patterns in the literature. The At2g41000 promoter-GUS fusion described by Tiwari et al. [16] was able to drive expression in the endosperm and the embryo sac but was not expressed in pollen. Clarification by semiquantitative RT-PCR was attempted to compare the GUS expression pattern with the endogenous transcript but the authors were unable to detect At2g41000 transcript in any tissue. Expression of this gene in online datasets is even more ambiguous with no signal apparent in dissected ovules and 1 DAP seed but expression in flower buds, roots, and leaves in the Goldberg Lab *Arabidopsis thaliana* Genechip Database (http://estdb.biology.ucla.edu/genechip) and only pollen expression being evident in the AtGenExpress Genechip developmental series [60].

A study into abscisic acid (ABA) synthesis has also identified an endosperm-specific 9-cis-epoxycarotenoid (*NCED6*) that cleaves cis-epoxycarotenoids in the first specific step of ABA biosynthesis. *NCED6* was found to be specific to the endosperm by promoter-GUS, promoter-GFP, and *in situ* hybridization [63] and is confirmed by the inclusion of NCED6 in our endosperm expressed list (Table 3). Expression constructs were also made for a second gene, *NCED9* However, despite using over 3 kb of upstream sequence, neither GUS nor GFP could be observed in the seed. This was inconsistent with RT-PCR data from RNA obtained from manually dissected seed and *in situ* hybridization, both of which measure the abundance of the endogenous transcript directly [63]. Comparisons of endogenous gene expression and reporter activity suggest that the upstream sequences used to drive reporter expression do not necessarily confer expression patterns identical to those of the endogenous gene as assessed by other methods such as *in situ* hybridization or RT-PCR [16, 58, 63].

Another endosperm marker that was consistent with our endosperm-expressed list is *SUC5* (Table 3). The *SUC5* gene encodes a sucrose transporter that is thought to contribute to the nutrition of the filial tissues during seed development. Baud et al. [64] examined the expression of the *SUC5* promoter using both GUS and GFP reporter constructs and linked the expression patterns to expression of the endogenous transcript by *in situ* hybridization studies. *SUC5* expression was found to be endosperm-specific, and at 4 DAP (early to mid heart stage) the expression is changing from localization in the embryo-surrounding region to a more general endosperm expression [64].

The endosperm-expressed list also contains three components of the fertilization independent seed (FIS) polycomb group complex; MEDEA (MEA), FIS2, and FIE (Table 3). Loss of function mutations of these components of the FIS-complex causes autonomous onset of cell division in the central cell without fertilization. Promoter-GUS lines indicate that these three genes are strongly transcribed in the central cell prior to fertilization. After fertilization, only the maternal alleles of these genes appear to be expressed in the endosperm and persist in the free nuclear endosperm until cellularization [65, 72]. As the endosperm cellularizes at approximately 5 DAP, GUS activity from MEA and FIS2 reporter constructs decline, whereas FIE promoter-GUS activity persists and can be observed in both the embryo and the cellularized endosperm [65]. However, MEA expression analysis by *in situ* hybridization and RT-PCR from manually dissected seed compartments indicate that MEA expression also occurs in the embryo at heart stage [26]. Interestingly, the transcripts of *MEA* and *FIE* have relatively low fold changes in our LM endosperm data (Table 3), which is consistent with *MEA* and *FIE* expressions in additional tissues within developing siliques [26, 65].

The FIS complex has been shown to repress the MADS-box gene *PHERES1* (*PHE1*). In contrast to the *FIS* genes described above, *PHE1* is not expressed in the central cell prior to fertilization and appears to be only expressed from the paternal genome after fertilization. *PHE1* promoter-GUS reporter lines indicated that *PHE1* expression is expressed in very early embryos and the endosperm [73]. At heart stage however, *PHE1* expression becomes restricted to the chalazal region of the endosperm, an expression pattern that is consistent with the high differential described by *PHE1* in our endosperm-expressed list. Another endosperm marker that describes a high differential in our endosperm list is *FWA* Although the function of *FWA* is unknown, its expression has been characterized using both reporter construct and *in situ* hybridization and has a similar expression to *FIS2*. *FWA* expression in the endosperm also appears to be solely from the maternal allele [19]. Imprinting in plants has been hypothesized to be an endosperm-specific phenomenon and our list of endosperm-expressed genes includes all confirmed imprinted genes in *Arabidopsis*, that is, *PHE*, *MEA*, *FIS2*, *FWA* [19, 26, 62, 73, 74], and the likely imprinted gene *FIE* [75] (Table 3).

In summary, our microarray analysis using endosperm-derived RNA identified approximately 2 700 genes with putative endosperm expression at 4 DAP using a 2-fold cutoff (Supplementary Table 1). The presence of known endosperm marker genes in this gene list indicates that our data is reliable.

## 4. Conclusions

With the extensive molecular resources available for *Arabidopsis* and established methods for RNA amplification, LM represents an attractive tool capable of providing high-resolution expression analysis and enables novel insights into the cellular processes that occur during early seed development. Here, we have established methods for the generation of expression profiles from specific seed tissues and have shown that despite some limitations, global RNA amplification from 50 ng of endosperm total RNA can generate high-quality expression data. This data should provide a useful resource for laboratories interested in early seed development.

## Supplementary Material

Supplementary Table 1 provides a list of approximately 2700 loci preferentially expressed in 4 DAP endosperm compared to 4 DAP silique. The loci were identified using a 2-fold cutoff and a *P* value of < .05 calculated from four hybridizations.

## Figures and Tables

**Table 1:** Amount of starting RNA and subsequent yields after amplification.

	Starting^(a)^ 1st round (*μ*g)	Average yield 1st round (*μ*g)	Starting^(c)^ 2nd round (*μ*g)	Average yield 2nd round (*μ*g)

Unamplified	30	—	—	—
IVT labelling	5	107^(c)^	—	—
2-round IVT	0.05	5.4^(c)^	2.7	103^(c)^
PCR	0.05	10^(b)^	—	—
LM samples^(d)^ (2-round IVT)	0.041	2.0^(c)^	0.5	51^(c)^

^(a)^ total RNA.

^(b)^ cDNA.

^(c)^ aRNA.

^(d)^ A mean yield value is included for comparison generated from seed coat, endosperm, and embryo amplifications.

**Table 2:** (a) The number (and %) of unique 2-fold differentials identified with each target preparation regime. (b) The number of transcripts identified as being differentially expressed (based on 1.5- and 2-fold cutoffs) using the mean Log_2_ ratio from duplicate hybridizations for each treatment.

(a)				
	Unamplified	IVT	2IVT	PCR

Unique x2	165 (7%)	488 (12%)	177 (6%)	104 (12%)

(b)				
		
Early	Early		90% late	90% late
x2	x1.5		x1.5	x2

1016	3306	Unamplified^(5)^	2617	1473
		(21 603)		

2015	4806	IVT	3628	2180
		(21 910)		

1343	3521	2IVT	3237	1703
		(20 966)		

246	1460	PCR	1625	601
		(21 367)		

^(a)^ The number of genes represented in the data for each method of target preparation after trimming out low intensity and flagged spots.

**Table 3:** Distribution of imprinted genes and endosperm, embryonic, and seed coat markers in the LM endosperm array data.

Locus	Description	Notes	Reference	Fold change by array^(a)^	Fold change by qPCR^(b)^

At1g65330	PHERES1 (PHE1)	Endosperm expressed/imprinted	[62]	5.48 0.00	—
At4g25530	Homeodomain protein (FWA)	Endosperm marker	[19]	5.40 0.00	—
At3g24220	9-cis-epoxycarotenoid dioxygenase (NCED6)	Endosperm marker	[63]	4.73 0.00	—
At3g26790	Transcriptional regulator (FUS3)	Embryo marker	[58]	4.49 0.00	4.36 ± 0.49
At1g71890	Sucrose transporter (SUC5)	Endosperm marker	[64]	4.49 0.00	—
At1g21970	CCAAT-box binding factor HAP3 homolog (LEC1)	Embryo and endosperm expressed	[57]	3.58 0.00	—
At2g35670	Fertilization-dependent seed 2 (FIS2)	Endosperm marker	[65]	3.55 0.01^(c)^	—
At1g02580	MEDEA (MEA)	Endosperm expressed/imprinted	[26]	3.34 0.00	—
At1g48630	WD-40 repeat auxin-dependent protein (ARCA-like)	Embryo and endosperm expressed	[53]	2.51 0.00	2.50 ± 0.38
At3g19160	tRNA isopentenyl transferase-related (IPT8)	Endosperm marker	[61]	2.17 0.00	—
At3g20740	Fertilization-dependent endosperm (FIE)	Endosperm expressed/imprinted	[65]	2.03 0.00	—

Recommended cutoff value				2.00		
At5g16780	Expressed protein	Embryo marker	[53]	1.72 0.01	1.61 ± 0.35
At4g09820	bHLH protein (TT8)	Seed coat marker	[55]	1.47 0.03	1.66 ± 0.03
At1g15750	Expressed protein	Embryo marker	[53]	1.26 0.16	—
At2g21320	CONSTANS B-box zinc finger family protein	Embryo marker	[53]	−1.16 0.08	—
At2g02760	Ubiquitin-conjugating enzyme 2 (UBC2)	Embryo marker	[53]	−1.18 0.08	—
At5g43810	PINHEAD translation initiation factor (ZWILLE)	Embryo marker	[66]	−1.28 0.07	—
At1g61720	Dihydroflavonol 4-reductase (BANYULS)	Seed coat marker	[56]	−1.32 0.04	—
At1g73590	Auxin transporter (PIN1)	Embryo marker	[67]	−1.39 0.31	—
At1g04550	Auxin-responsive protein IAA12 (BODENLOS)	Embryo marker	[68]	−1.39 0.07	—
At4g37750	Ovule development protein aintegumenta (ANT)	Embryo marker	[69]	−1.43 0.09	—
At5g17430	Ovule development protein, putative	Embryo marker	[53]	−1.54 0.14	—
At2g34650	Protein kinase (PINOID/PID)	Embryo marker	[70]	−1.82 0.01	—
At1g19850	Auxin response factor (MONOPTERUS)	Embryo marker	[68]	−4.72 0.00	—
At2g01420	Auxin transport protein (PIN4)	Embryo marker	[71]	−10.09 0.00	—
At1g70940	Auxin transport protein (PIN3)	Embryo marker	[71]	−10.95 0.00	—

^(a)^ Value to the right represents *P* value.

^(b)^ Value to the right represents SE when *n* = 3.

^(c)^ Normalized without interpolation as signal was very low.
